# Feasibility and Usage of a Virtual Assistant Device in Cognitively Impaired Homebound Older Adults

**DOI:** 10.1177/07334648251314284

**Published:** 2025-01-10

**Authors:** Matthew Nunez, Prisha Patel, Lindsey Ulin, Leslie Kian, Martin Cominsky, Jason Burnett, Jessica L. Lee

**Affiliations:** 1 McGovern Medical School, 12340 The University of Texas Health Science Center at Houston, Houston, TX, USA; 2 Department of Epidemiology, Human Genetics, and Environmental Sciences, School of Public Health, 12340 The University of Texas Health Science Center at Houston, Houston, TX, USA; 3 Department of Medicine, 1861 Brigham and Women’s Hospital, Boston, MA, USA; 4 496762 Meals on Wheels Greater Houston, Interfaith Ministries for Greater Houston, Houston, TX, USA; 5 Healthcare Data and Analytics Association, Salt Lake City, UT, USA; 6 Joan and Stanford Alexander Division of Geriatric and Palliative Medicine, McGovern Medical School, 12340 The University of Texas Health Science Center at Houston, Houston, TX, USA

**Keywords:** technology, home- and community-based care and services, cognitive function, virtual assistant device, homebound older adults

## Abstract

Social technology in older adults can improve self-rated health; however, there can also be difficulties using it. Our study aimed to evaluate the feasibility and acceptance of virtual assistant device (VAD) use in cognitively impaired homebound older adults. 52 newly referred Meals on Wheels clients aged 60 and older were recruited for a three-phase study: 6 weeks of meals alone (control), followed by 6 weeks of meals+Alexa Echo Show 8 (AES8) basic usage, and lastly 6 weeks of meals+AES8 advanced usage. Technology acceptance with the AES8 was significantly higher by the end of the study and participants anecdotally enjoyed playing music, setting reminders, and accessing spiritual content. There were also associations with improvements in memory, depression, and gait speed, despite no specific health programming. Thus, we believe use of VADs for cognitively impaired homebound older adults have future potential to benefit their cognitive and physical health. **ClinicalTrials.gov ID:** NCT04581317. **Impact Statement:** We certify that this work is novel because we were able to highlight improvements in the feasibility of use of a voice-activated virtual assistant device, technology acceptance, and some health indicators for underserved, cognitively impaired homebound older adults.

**What this paper adds**

• Cognitively impaired homebound older adults are a vulnerable population with high rates of morbidity, disability, and institutionalization.
• Our study has shown that they are able and willing to use voice-activated virtual assistant devices with potential improvements in their cognitive and physical health.
• More widespread use of these devices with appropriate technical support and health-specific programming could help these underserved older adults more successfully age in place.

**Applications of study findings**

• Cognitively impaired homebound older adults are able and willing to successfully use voice-activated virtual assistant devices.
• Use of the Amazon Alexa Show 8 led to potential improvements in depression, memory, and gait speed despite no specific health programming.
• In the future, use of cognitive and physical health-specific applications with virtual assistant devices may allow cognitively impaired older adults to age in place more independently.

## Introduction

Globally, technology is increasingly playing a central role in health and wellness across the lifespan. There has been a notable shift in the healthcare technologies which were once focused on specific diagnostics and medical treatments as they are now also being designed for use by non-professional consumers of all ages to support their self-management of healthy behaviors through smart technology and applications (Kratzke & Cox, 2012). While older adults are growing more accustomed to technology and studies have demonstrated positive health effects, it is unclear if these studies generalize to homebound older adults with the highest basic social needs (Andrews et al., 2019).

According to the 2020 U.S. Census, adults 65 years and older make up 16.8% of the population. This represents a population growth of nearly 1000% since 1920, making older adults the fastest-growing age group in the U.S. (Caplan, 2023). Homebound older adults (i.e., individuals unable to leave their home without the assistance of another person), largely due to cognitive or physical impairments, made up 13% of the U.S. population in 2020—more than doubling since 2013 (Ankuda et al., 2021). Given their higher level of supportive needs, homebound older adults may benefit from technology with potential to increase their independence in meeting their own needs and improve their emotional, cognitive, and physical health. Virtual assistant devices (VADs) are potential resources for homebound older adults, but many older adults have expressed unfamiliarity with VADs such as Amazon and Google devices, which are often gifted or given to them (Orlofsky & Wozniak, 2022). Many are never used at all because of limited awareness and understanding of the device features and service applications. Alternatively, usage may be minimal due to frustrations with technical limitations or the inability of the VADs to understand older adult speech patterns, even with voice capabilities. Older adults may also be subject to some of the negative ramifications that can come with technology use. Consequences include being persuaded by false information on the internet and social media or being victims of internet scams (Leist, 2013).

Despite these reservations, many older adults can see the potential benefits of technology such as VADs. One study showed that older adults are open to the use of technology to improve mental health and reduce the burden on others, such as their caregivers (Andrews et al., 2019). Others have found that older adults frequently use the internet, with 71% going online at least once per day (Smith, 2014). Additionally, nearly 94% of older participants in one study indicated that they had a “good” or “excellent” experience using a tablet, and 66.6% indicated they would use tablets in the future (Vaportzis et al., 2017). Thus, older adults may be inclined to use such devices if given the proper education on their use and potential features. In addition, these studies did not include homebound older adults or older adults with cognitive impairment, representing a missed opportunity to learn if VADs could function as supportive devices to help them meet their needs and promote better health.

We conducted a study evaluating whether VADs, specifically the Amazon Alexa Show 8 (AES8), could be appropriately utilized and possibly benefit the health of cognitively impaired homebound older adults enrolled in Meals on Wheels (MOW). We primarily focused on feasibility and acceptance of the AES8 by our homebound older adults with secondary measures of social determinants of health, cognitive health, and physical/functional status.

## Methods

### Sample Selection and Recruitment

This protocol was approved by The University of Texas Health Science Center at Houston’s Institutional Review Board (HSC-MS-20–0857). Homebound older adults were recruited from the Meals on Wheels for Greater Houston and Galveston County (MOWGH) program, administered by Interfaith Ministries of Greater Houston. MOWGH is one of the largest meal providers in Texas, delivering nutritious meals to the homes of over 5000 adults aged 60 and older. Their clients are primarily from underserved backgrounds: 44% African-American/Black, 16% Hispanic/Latino, 24% Caucasian/White, and 1% Asian/Pacific Islander.

Study participants were recruited by the MOWGH assessment team at the time of their initial client intake. Inclusion criteria were: 1) adults age 60 or older who were new to MOW, 2) had cognitive impairment as reported in the Mental Health Screening (MHS) assessment section of the MOWGH intake form (Supplemental Figure 1), 3) lived alone or with a caregiver, and 4) were medically stable. Screened participants were excluded if they had a pre-diagnosed terminal illness, were unable to ambulate, and/or were unable to use their upper extremities.

### Study Design

All consented participants were evaluated in their homes, at their baseline visit, by a medically licensed geriatrician to obtain a brief social and medical history for purposes of screening for inclusion/exclusion criteria. Demographic data, including age, race, gender, marital status, and living situation, were collected.

All participants were enrolled in the 3-phase intervention. Phase 1 consisted of 6 weeks of meals only as a control period, Phase 2 was 6 weeks of meals + AES8 basic usage, and Phase 3 was 6 weeks of meals + AES8 advanced usage (Supplemental Figure 2).

In Phase 1, MOWGH drivers delivered enough frozen meals to cover lunch for 5 days and breakfast for 7 days (12 meals weekly) for 6 weeks. The meals were created with the guidance of a registered dietician and met the Dietary Reference Intakes (DRI) for older adults set forth by the Food and Nutrition Board of the Institute of Medicine (Trumbo et al., 2002). Study staff called the participants twice weekly to ask 5 questions about their health, mood, and meal consumption. At the end of the control period, in-person visits were made to the participants’ homes by a trained study staff member to take measures of mental and cognitive health and functional status.

In Phase 2, participants continued to have meal deliveries and the AES8 device was installed. The AES8 was chosen due to its larger screen, touchscreen interface, and voice-activated capabilities. Before installation, the AES8 device was configured to the most restrictive default privacy settings, including turning the listening voice analytics setting off. Participants were taught how to turn off the microphone with a single button and the camera lens shutter was closed. Participants were also given basic instructions for using the device and a one-page handout that included ways to use the voice-activated features. The study staff continued to call twice a week to ask the 5 questions and at the end of this 6 week period, a second in-person visit was made to collect study measures.

In Phase 3, participants continued to have meal deliveries and the AES8 was upgraded to provide a more active intervention by asking the twice weekly questions through the device instead of via phone calls from the study staff (Supplemental Figure 3). The participants were able to answer the questions verbally or by touch screen options displayed on the device. At the end of the final 6 weeks, the study team visited the participants’ homes to take study measures and collect anecdotal feedback about their most common uses for the device. Participants were allowed to keep the AES8 device on completion of the study.

### Study Measures

Feasibility was assessed through the number of participants recruited and retained throughout the 3 phases of the study. Acceptance was measured using the technology acceptance measure (TAM) after Phases 2 and 3. The TAM is based on 4 categories (perceived ease of use, behavioral intention, computer self-efficacy, and perceived enjoyment) with 5 questions: 1) I find the AES8 to be easy to use, 2) I find it easy to get the AES8 to do what I want it to do, 3) Given that I have access to the AES8, I intend to use it, 4) I could use the AES8 if I had no one around to tell me what to do as I go, and 5) I find using the AES8 to be enjoyable (Klimova & Poulova, 2018; Macedo, 2017; Mitzner et al., 2019; Peek, 2017; Venkatesh et al., 2003, 2008). Participants were asked to answer these questions on a Likert scale from strongly disagree to strongly agree (Supplemental Figure 4).

Mental and cognitive health were evaluated at all of the visits using the Center for Epidemiologic Studies Depression Scale (CES-D) to measure depression and the Montreal Cognitive Assessment (MOCA) to measure cognitive impairment (Abd Razak et al., 2019; Mohebbi et al., 2018; ). CES-D is a 20 question, self-reported Likert scale (0–3) for measuring depression with a possible score range of 0–60 with higher scores indicating more depressive symptoms. It has an estimated internal consistency reliability of 0.89–0.91 for older adults. The MOCA is a memory test that is extensively used in older adults with a maximum score of 30 and a cutoff of 25 or below used to indicate cognitive impairment. The MOCA has good internal consistency reliability of 0.82.

Functional status was evaluated at all of the visits using Activities of Daily Living (ADL) and Instrumental Activities of Daily Living (IADL) (Fieo et al., 2011). ADLs were scored 0 for dependent or 1 for independent in six domains of bathing, dressing, toileting, transferring, continence, and feeding; while IADLs were also scored 0 or 1 in eight domains of telephone use, shopping, food preparation, housekeeping, laundry, transportation, medications, and finances. Frailty was measured by the Physical Frailty Phenotype (PFP) components of unintentional weight loss, weakness, poor endurance, slowness and low physical activity (Fried et al., 2001). Participants with no characteristics were categorized as robust, those with one or two characteristics were categorized as pre-frail, and those with three or more characteristics were considered frail. The Zarit Caregiver Burden Interview (ZCBI) was collected at all visits only for participants with caregivers (Whalen & Buchholz, 2009). ZCBI is a self-reported questionnaire that measures the impact of caregiving on a person’s life through the perceived impact of caregiving on the caregiver’s physical health, emotional health, social activities, and financial situation. The ZCBI is composed of 22 questions rated on a Likert scale, with a possible range of scores from 0 to 88. Higher scores indicated a greater caregiver burden. The internal consistency reliability is high (0.93).

### Statistical Analysis

Because this study was designed to evaluate feasibility of AES8 use and technology acceptance, a formal power analysis was not conducted. Data preparation was conducted by first checking impossible values to ensure the accuracy of the data. There were only less than 1% missing values in each time point, so pairwise deletion was used to treat the missing value. Next, reliability using Cronbach’s alpha was tested to confirm the inter-item consistency at baseline on the depression and caregiver burden instrument tools, and the results showed very strong reliability of the tools (Cronbach’s alpha = .907 and .918, respectively). Reliability using intra-class correlation (ICC) was used to evaluate the consistency of the repeated measures on the grip strength and gait. The results revealed very strong reliability among the three measures of the grip strength and among the two measures of the gaits at every time point (all ICCs > .90). Then, assumption tests (such as normality, outliers, and equality of variance, etc.) were performed to determine if the data met assumptions for the parametric analysis. Finally, correlations among all baseline measures showed that most baseline outcomes were not significantly correlated with each other, so univariate analyses rather than multivariate analyses were used.

The sample was characterized using descriptive statistics. Frequencies and percentages were reported on the categorical variables, and mean and standard deviation (SD) were reported on the continuous variables. Next, normality was assessed and bivariate analysis was conducted between demographic variables and baseline outcome measures to identify relevant covariates. Repeated measures analysis of variance (ANOVA) or analysis of covariance (ANCOVA) were conducted to compare continuous score changes over 4 visits. Grip strength was not normally distributed with a few outliers and caregiver burden had a small sample size, so Friedman nonparametric analyses and Wilcoxon signed rank nonparametric analyses were used to confirm the results. For the gait speed, only the participants who did not use an assistive device during the visit were included in the gait comparison, so a series of paired t-tests were used to compare every two visits. With the categorical outcomes, Marginal Homogeneity tests were conducted to compare every two visits on TAM, MOCA, PFP, and ZCBI. Cochran’s Q and McNemar’s tests were conducted to compare every two visits on ADL, IADL, and CES-D. All data were analyzed in SPSS v28 and *p* < .05 was used to determine statistical significance.

## Results

### Demographics

52 participants enrolled in the study, with 45 participants included in the data analysis as they completed at least 2 visits (Table 1). Most participants were females (71%) with an average age of 74 years. The majority of participants were African American (71%), while the rest were White/Caucasian (27%), and Hispanic (2%). A little over one-third of the participants were widowed (38%), followed by those who were married (24%) and divorced (18%). Participants mainly had associate degrees (24%), followed by high school diploma (20%) or completed Grades 9-12 (20%). Participant characteristics were similar between those enrolled and those retained for analysis, reflecting no discernable attrition bias.

**Table 1.** Demographic Characteristics.

Characteristics	Enrolled *(N* = 52)		Retained *(N* = 45)	
	*N*	*%*	*N*	*%*
Gender				
Female	37	71.2	32	71.1
Male	15	28.8	13	28.9
Ethnicity				
Non-Hispanic	50	96.2	43	95.6
Hispanic	2	3.8	2	4.4
Race				
African American	38	73.1	32	71.1
White/Caucasian	13	25.0	12	26.7
Hispanic	1	1.9	1	2.2
Marital status				
Widowed	20	38.5	17	37.8
Married	12	23.1	11	24.4
Divorced	10	19.2	8	17.8
Single/Never married	7	13.5	6	13.3
Separated	3	5.8	3	6.7
Educational level				
Graduate degree	4	7.7	4	8.9
Bachelor’s degree	4	7.7	3	6.7
Associates degree	13	25.0	11	24.4
Some college	5	9.6	5	11.1
High school diploma	9	17.3	9	20.0
Grades 9 to 12	13	25.0	9	20.0
Grades 6 to 8	4	7.7	4	8.9
**Age,** mean (SD)	74.02 (8.92)		73.80 (9.27)	

### Feasibility and Acceptance

#### Recruitment and Retention

**Figure 1.**
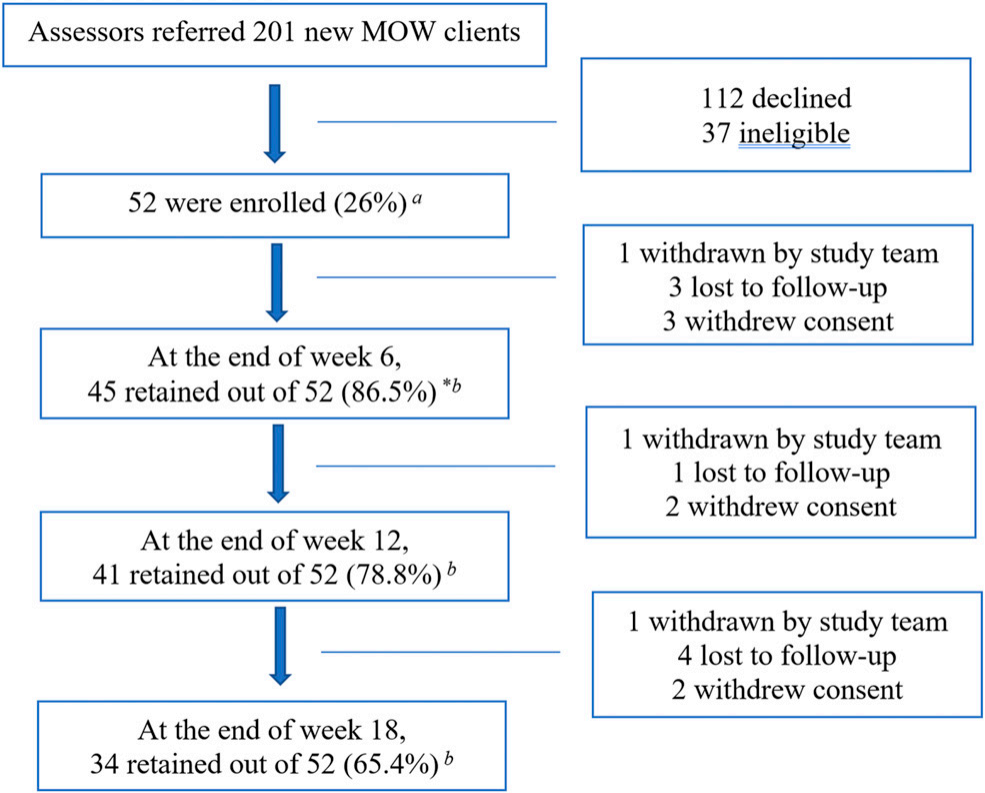
Study participant flowchart. *Included in the data analysis after completing 2 visits; ^*a*^Percentage in recruitment process; ^*b*^Percentage in retention.

201 new MOW clients were referred by the MOWGH assessors to the study team with 112 declining to participate (56%) **(**Figure 1). 37 potential participants (18%) were deemed ineligible after speaking with the study team, so 52 participants (26%) qualified and were enrolled into the study. After Phase 1, 7 dropped out of the study (87% retention) and another 4 participants dropped from the study by the end of Phase 2 (79% retention). After Phase 3, the number of participants who completed the entire study was 34 (65% retention), however 45 participants were included in the data analysis as they completed 2 visits.

#### Technology Acceptance Measure

**Figure 2.**
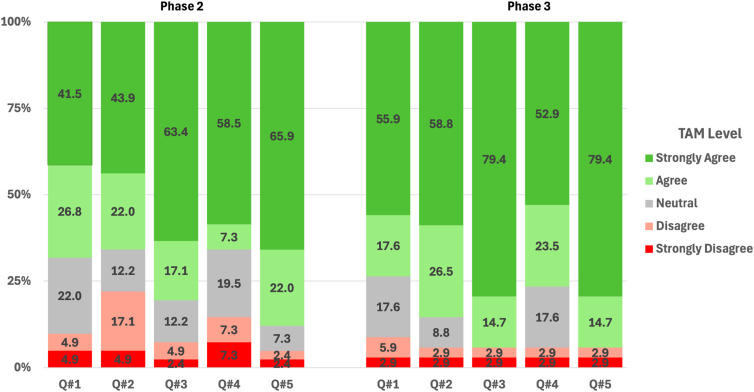
Technology Acceptance Measures. Percentages of satisfaction levels on five technology acceptance questions between Phase 2 and 3.

Five technology acceptance questions were asked to evaluate the usage and self-efficacy of AES8 at the end of Phase 2 and Phase 3 (Figure 2). Most participants agreed/strongly agreed at the end of Phase 2 that the AES8 was easy to use (TAM1: 68.3%), was easy to get the AES8 to do what they wanted (TAM2: 65.9%), they intended to use it (TAM3: 80.5%), they would use it if no one else was around (TAM4: 65.8%), and using it was enjoyable (TAM5: 87.9%). When comparing the Phase 3 to Phase 2 TAM, the results indicated a significant improvement in satisfaction in TAM2 (*MH* = 50.00, *p* = .008) and TAM3 questions (*MH* = 34.00, *p* = .046).

### Mental and Cognitive Health Outcomes

#### Depression

**Figure 3.**
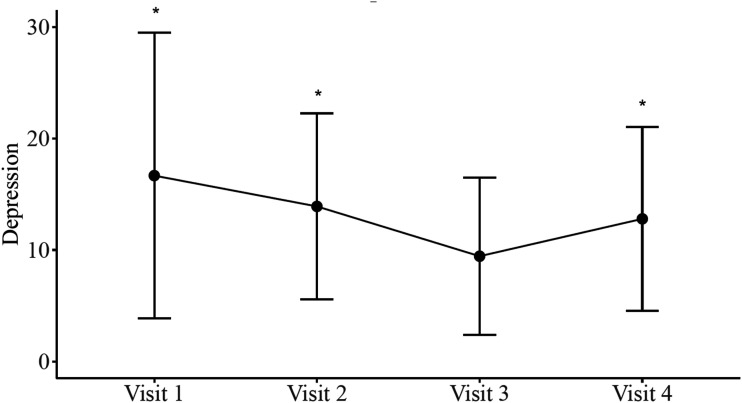
Center for Epidemiologic Studies Depression Scale. Depression scores over 4 visits. Higher scores indicate more severe depression. Values are mean ± SD. *, significant differences were seen in all other visits compared to visit 3 – basic AES8 (*p* < .05).

During preliminary analysis, we found that older age was moderately and significantly related to less depression (*r* = −.462, *p* = .001), so age was controlled for in the repeated measures ANCOVA. The results showed that overall the depression scores significantly decreased over time (*Huynh-Feldt’s F* = 3.546, *p* = .030) (Figure 3). More detailed analysis showed that depression scores were significantly lower after Phase 2 when the AES8 was first installed (Visit 3) (*M* = 9.22, *SD* = 7.07) in comparison to baseline (Visit 1) (*M* = 16.68, *SD* = 12.81), Phase 1 of meals only (Visit 2) (*M* = 13.91, *SD* = 8.33), and Phase 3 with advanced AES8 usage (Visit 4) (*M* = 12.79, *SD* = 8.25), suggesting that the meals + AES8 basic usage intervention may alleviate depression (all *p*s < .005).

#### Memory

**Figure 4.**
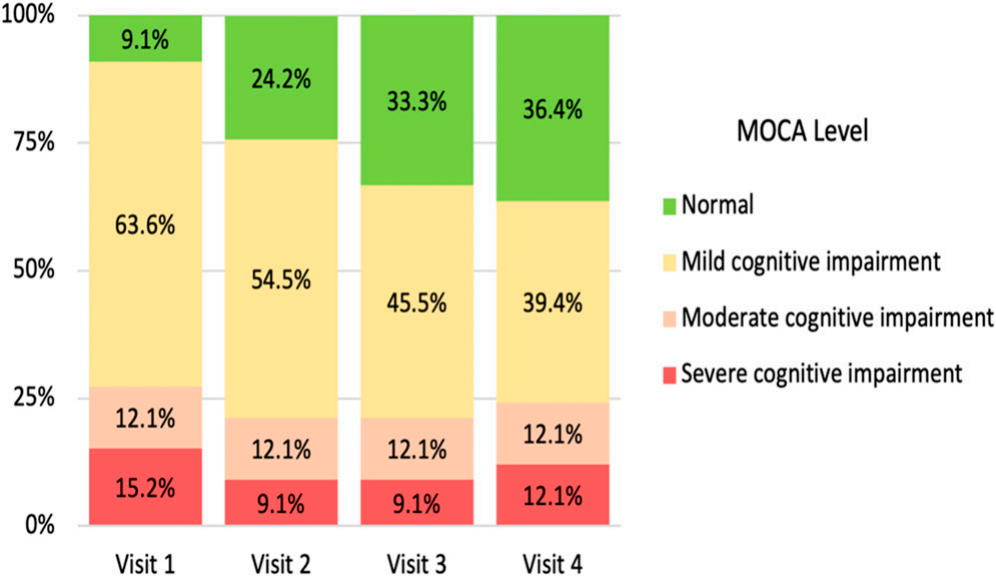
Montreal Cognitive Assessment. Percentages of participants with varying cognitive levels over 4 visits.

Cognitive function was measured by MOCA with higher scores indicating better cognitive function. Similar to depression, we found that older age was moderately and significantly related to worse cognition (*r* = −.409, *p* = .005), so age was controlled for in the repeated measures ANCOVA. The results showed that there were no significant differences across all visits (*F* = .449, *p* = .718). However, when the cognitive scores were categorized into four levels, the results revealed cognitive level improvements over time (all *p*s < .05) (Figure 4). Greater proportions of participants had normal cognitive function in Visit 2 than in Visit 1 and in Visit 3 than in Visit 2, whereas proportions of participants with mild cognitive impairment declined over time.

### Physical and Functional Outcomes

#### Functional Status

**Figure 5.**
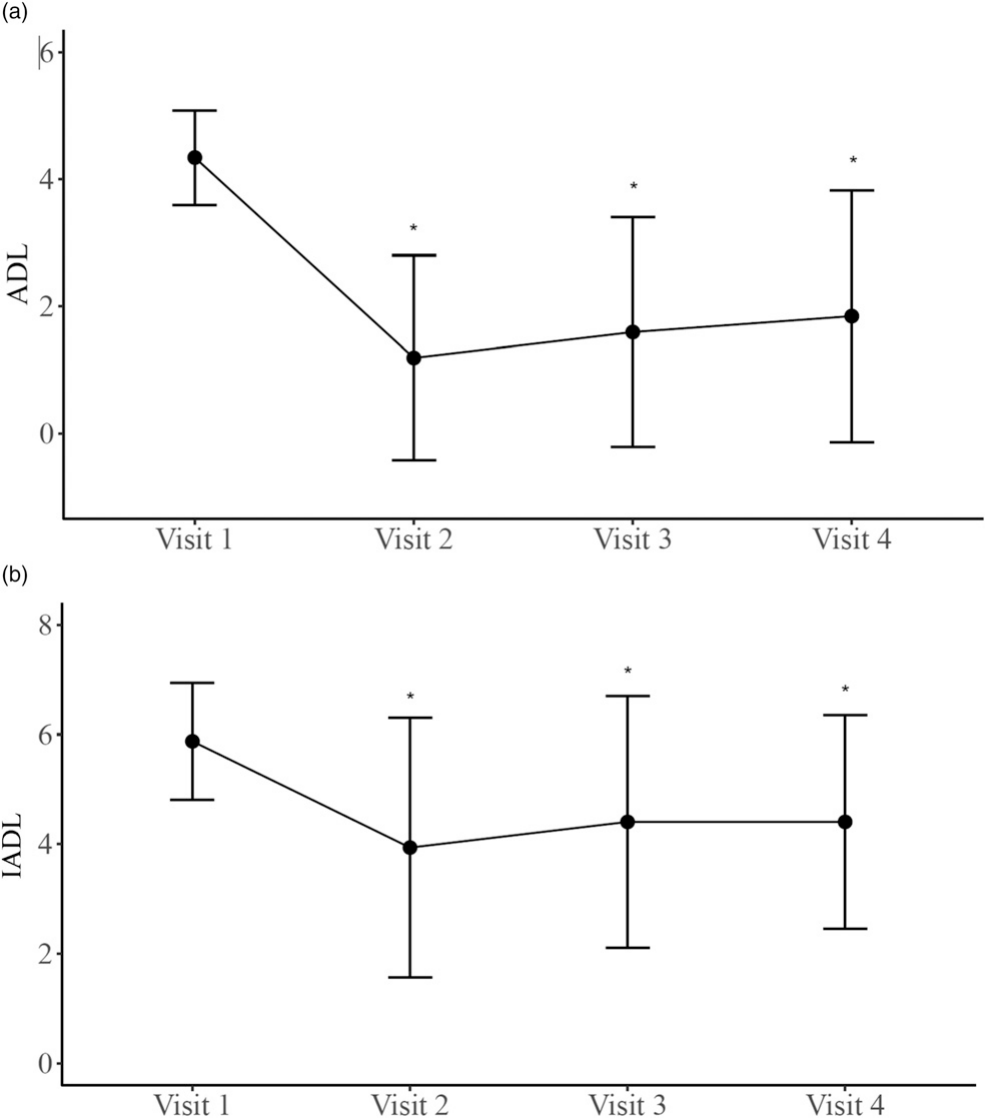
Counts of (a) Activities of Daily Living (ADL) and (b) Instrumental Activities of Daily Living (IADL) over 4 visits. Higher scores indicate more severe impairments. Values are mean ± SD. *, significant differences were seen in all other visits compared to visit 1 (*p* < .05).

Both ADL and IADL scales were used to assess participants’ ability to perform a daily task. The number of activities unable to be performed was counted, and overall these significantly changed over time (*F* = 36.770 and *p* < .001, *F* = 13.706 and *p* < .001, respectively) (Figure 5). A more in-depth analysis showed that the number of daily living activities that could not be performed was significantly reduced at visits 2−4 from Visit 1 for both ADL and IADL (all *p*s < .001). However, there were no significant differences across Visits 2, 3, and 4.

#### Physical Frailty

African Americans had significantly lower PFP scores than White/Hispanic participants (*p* = .035, Cohen’s *d* = .715), so race was controlled in the repeated measures ANCOVA. The results showed that there were no significant differences in the PFP scores across the visits (*F* = 1.191, *p* = .318). As the PFP was categorized into robust, pre-frail, and frail, no significant level changes were found over time either (all *p*s > .05).

However, additional analysis done on one of the components of the PFP, gait speed, showed significantly faster times at visit 3 (*M* = 7.03, *SD* = 2.25) as compared with the scores at visit 2 (*M* = 8.13, *SD* = 2.94) (*p* = .044, *Cohen’s d* = .417) (Supplemental Figure 5). No significant changes were found from visit 1 to visit 2 or from visit 3 to visit 4.

#### Caregiver Burden

In our study, only a small number of participants had caregivers (*N* ≤10), so nonparametric analyses were used to compare burden in each pair of visits. The results showed that caregiver burden was only significantly decreased from Visit 1 to Visit 4 (*Z* = 1.997, *p* = .046), but significance was not found in Visits 2 and 3 as compared with Visit 1 (Supplemental Figure 6). Given the small sample size, the non-statistical significance was likely due to low power and large variation, so the effect size *r* was reported as well. The results suggest that the caregiver burden scores were decreased from Visit 1 to Visit 2 with moderate effect size (*r* = .47), from Visit 1 to Visit 3 with moderate effect size (*r* = .41), and from Visit 1 to Visit 4 with large effect size (*r* = .82). It was also found that the burden was reduced from Visit 2 to Visit 4 with moderate-large effect size (*r* = .48).

## Discussion

Our results indicate that use of a VAD, specifically the AES8, is feasible in cognitively impaired and socioeconomically underserved homebound older adults and may have benefits for their health. Our participants reported good technology acceptance with higher acceptance associated with more technology exposure. Over time, participants reported more ease at getting the AES8 to do what they wanted and higher intentions of using the device in the future. Anecdotally, they appreciated being able to find and play music, set reminders, and access spiritual content.

Individuals who are older, less educated, poorer, and/or members of an ethnic minority group are up to five times less likely to have access to digital health information than those who are younger, more highly educated, or White/Caucasian (Tappen et al., 2022). However, despite some technical issues, our cognitively impaired homebound older adults showed significant improvements in their ability to use the AES8 device through both basic and advanced phases. We posit that this may be due to the simplicity of the AES8 device interface (both touchscreen and voice-activated), technical assistance with installation, and available troubleshooting by the study team.

In terms of mental health and cognition, our participants had improved depression scores with the use of the AES8, over the delivery of meals alone. In recent years, social technology and particularly social media, has faced scrutiny for its association with increased rates of depression among certain users (Ghaemi, 2020). However, our research contributes a nuanced perspective by highlighting the potential positive impact of VADs on depression among older adults. Given the well-established, literature-supported link between social isolation and increased risk of depression, our findings suggest the importance of exploring innovative solutions, such as VADs, for possible social interactions. Additionally, cognition appeared to change favorably for most participants over time which may have been related to Alexa engagement. Being able to set reminders for tasks like taking medications may allow cognitively impaired older adults to retain some independence and has been shown to improve cognitive function (Hackett et al., 2022).

For the small number of participants who had caregivers, we did find decreases in caregiver burden from baseline to end of study with moderate-large effect size. It may be that AES8 basic usage helped with caregiver burden but the advanced task of answering biweekly questionnaires was more burdensome. With a larger sample size of caregivers, caregivers could perhaps be trained to utilize AES8 features for daily information retrieval, task reminders, and enhanced social interaction, promoting more independence for the patient and reducing reliance on caregiver assistance.

Functionally, we found that improvements in ADLs/IADLs were mostly related to meal delivery and less related to AES8 usage. Surprisingly, gait speed increased after the addition of the basic AES8. While we did not emphasize specific exercise programs for the participants, it is possible that they were motivated to move more with the device. This suggests that further exploration of incorporating specific exercise programs targeting balance, mobility, and strength with the VAD may be beneficial to this population.

Our study findings should be considered in the context of certain limitations. First, this was a quasi-experimental design that did not use a conventional control group. There may also be limitations in generalizability due to small sample size and possible sampling bias as over 50% of MOW clients refused participation. Despite these limitations, the findings raise the possibility of using voice-activated technology to support positive health in homebound older adults and should be further explored.

Our study contributes to the understanding of technology adoption among a population less familiar with VADs, shedding light on potential disparities in access and utilization. Our research reveals that providing hands-on assistance with installation and easy access to technological support for troubleshooting helps cognitively impaired homebound older adults successfully use technology such as VADs. Future studies evaluating remote installation and technological support for VADs could further expand to rural populations. Specific applications such as exercise programming or cognitive games could enhance improvements in their physical and cognitive health. By addressing specific needs through tailored interventions and support mechanisms, there are promising opportunities to bridge the technological gap and unlock the potential that technology holds for enhancing the lives of cognitively impaired homebound older adults.

## Supplemental Material

Supplemental Material - Feasibility and Usage of a Virtual Assistant Device in Cognitively Impaired Homebound Older Adults Supplemental Material for Feasibility and Usage of a Virtual Assistant Device in Cognitively Impaired Homebound Older Adults by Matthew Nunez, Prisha Patel, Lindsey Ulin, Leslie Kian, Martin Cominsky, Jason Burnett, and Jessica L. Lee in Journal of Applied Gerontology
