# Supplementary material for: Feasibility and Usage of a Virtual Assistant Device in Cognitively Impaired Homebound Older Adults
Source: J Appl Gerontol. 2025 Jan 10;44(10):1651–60. doi: 10.1177/07334648251314284 (PMC12241460; doi:10.1177/07334648251314284)

SUPPLEMENTAL MATERIALS

Supplemental Figure 1. Meals on Wheels Mental Health Screening Form

Texas Department of Aging and Disability Services
Area Agency on Aging
AAA Consumer Needs Evaluation – Page 2

Area Agency on Aging

Consumer Name: ________________________

Consumer Number: ___________________

Assessment Date: ______________________

Service Arrangement
C = Caregiver
P = Service-will be purchased by AAA.
A = Other agency-non-AA vendor is providing the service.
N = Not applicable to this consumer.
S = Self

	Texas Score	NAPIS ADL/IADL	NAPIS Count	Scoring/Service Arrangement
**II. Mental Health Screening**				
**21.** During the last month, have you been bothered by having little interest or pleasure in doing things, or have you often felt down, depressed, or hopeless?				Scoring for question 21:<br>0 = if the answer is "No" to question 21<br>1 = if the answer is "Yes" to 21 and "No" to questions 22-25<br>2 = If the answer is "Yes" to 21 *and* "*Yes*" to at least one of questions 22-25<br>3 = If the answer is "Yes" to 21 *and* "Yes" to two or more of questions 22-25
**III. Mental Health Assessment –**<br>**If the answer is YES to Question 21, continue.**<br>**Otherwise, SKIP to section IV.**				
In the last two weeks, most of the day, nearly every day:				Based on Consumer's perception of self:
**22.** … have you had problems sleeping?				Answer "No" or "Yes" for this question
**23.** … have you lost the ability to enjoy things that once were fun?				Answer "No" or "Yes" for this question
**24.** … do you feel that you have little value as a person?				Answer "No" or "Yes" for this question
**25.** … have you had a significant change in your appetite?				Answer "No" or "Yes" for this question
**Mental Health Assessment Score (II & III)**				
**IV. Cognition**				
**A. Self-Evaluation**				
**26.** During the last 2 weeks, on how many days have you had trouble concentrating or making decisions? (Based on Consumer's perception of self.)				0 = Not at all<br>1 = Occasionally, a couple of times<br>2 = Frequently, more than a couple times, but not every day<br>3 = Every day
**B. Third Party Observation**				
**27.** Does the consumer have the ability to make decisions independently? (Based on someone's observation of the Consumer).				0 = Makes consistent and reasonable decisions independently<br>1 = Makes simple decisions without assistance<br>2 = Makes poor decisions, needs cues/supervision for most decisions<br>3 = Severely impaired, rarely makes own decisions.
**28.** Does the consumer appear to have short-term memory impairment? (Based on someone's observation of the Consumer.)				0 = No<br>1 = Has some short-term memory problems & can perform task for self with occasional reminders<br>2 = Has lapses resulting in frequently not performing task even with reminders<br>3 = Has memory lapses resulting in inability to perform routine tasks on a daily basis

Supplemental Figure 2. Study Phases

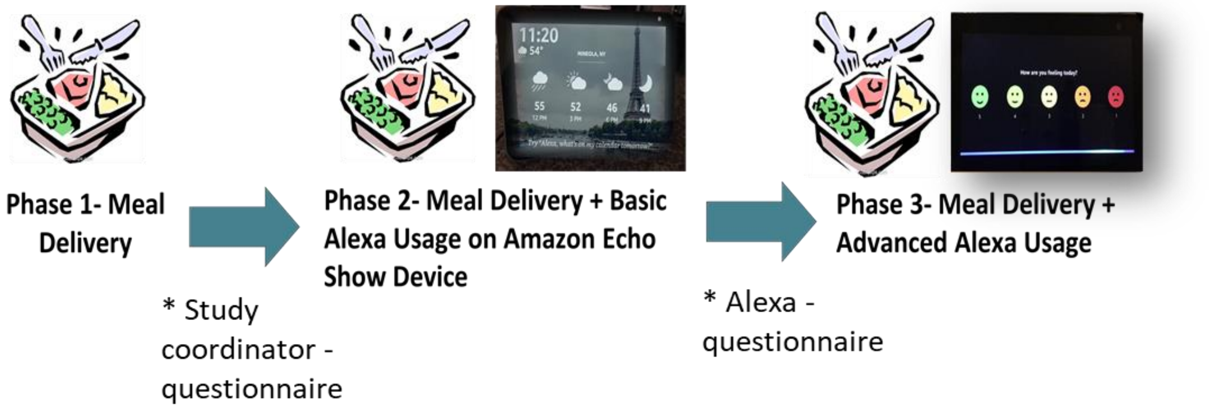

Supplemental Figure 3. Advanced Alexa Echo Show 8 Questionnaire

QUESTION(S) TO BE ASKED	Day of the Week:	Day of the Week:
1. How are you feeling today?	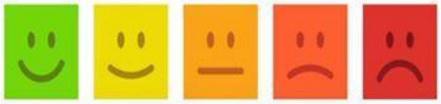	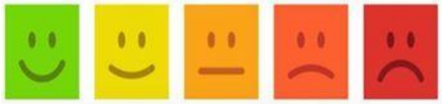
2. How has your overall health been?	☐ Excellent ☐ Very Good ☐ Good ☐ Fair ☐ Poor	☐ Excellent ☐ Very Good ☐ Good ☐ Fair ☐ Poor
3. Has poor physical or mental health kept you from doing your usual activities, such as self-care?	☐ Yes ☐ No	☐ Yes ☐ No
4. Have you been feeling as though you are not getting enough rest or sleep?	☐ Yes ☐ No	☐ Yes ☐ No
5. Have you had poor appetite or have not been eating all of your meals?	☐ Yes ☐ No	☐ Yes ☐ No

Supplemental Figure 4. Technology Acceptance Measure

Perceived Ease of Use	I find the Alexa Echo Show to be easy to use.	Strongly Agree, Agree, Neutral, Disagree, Strongly Disagree
	I find it easy to get the Alexa Echo Show to do what I want it to do.	Strongly Agree, Agree, Neutral, Disagree, Strongly Disagree
Behavioral Intention	Given that I have access to the Alexa Echo Show, I intend to use it.	Strongly Agree, Agree, Neutral, Disagree, Strongly Disagree
Computer Self-Efficacy	I could use the Alexa Echo Show if I had no one around to tell me what to do as I go.	Strongly Agree, Agree, Neutral, Disagree, Strongly Disagree
Perceived Enjoyment	I find using the Alexa Echo Show to be enjoyable.	Strongly Agree, Agree, Neutral, Disagree, Strongly Disagree

Supplemental Figure 5. Gait speed in seconds over 4 visits. Shorter time indicates faster speed. Values are mean ± SD. *, significant difference from visit 2.

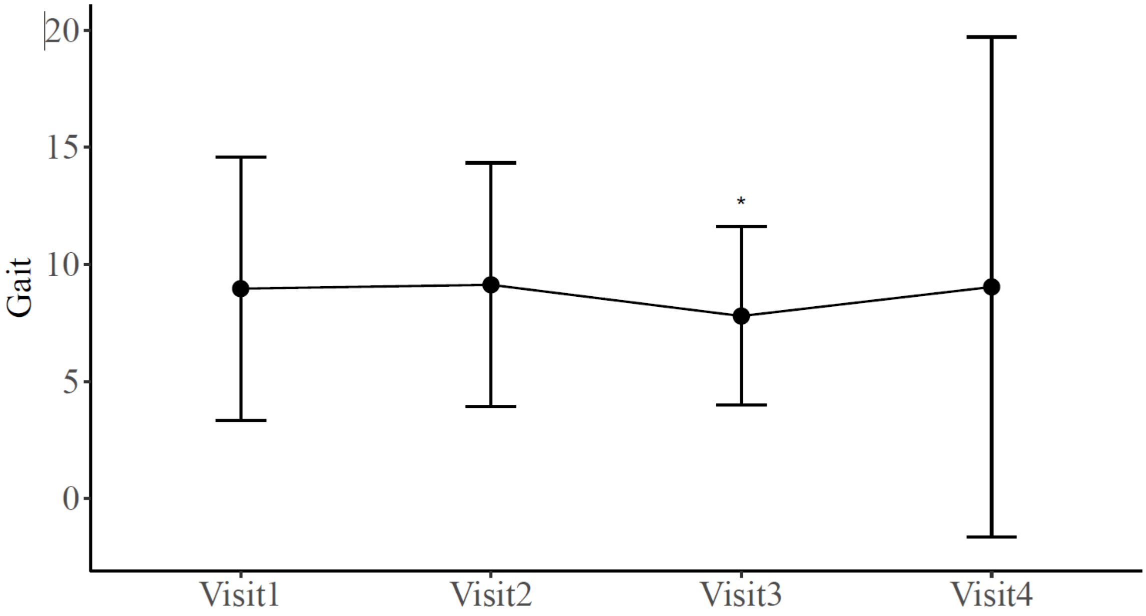

Supplemental Figure 6. Caregiver burden scores over 4 visits. Higher scores indicate more severe caregiver burdens. Values are mean ± SD. *, significant difference from visit 1.

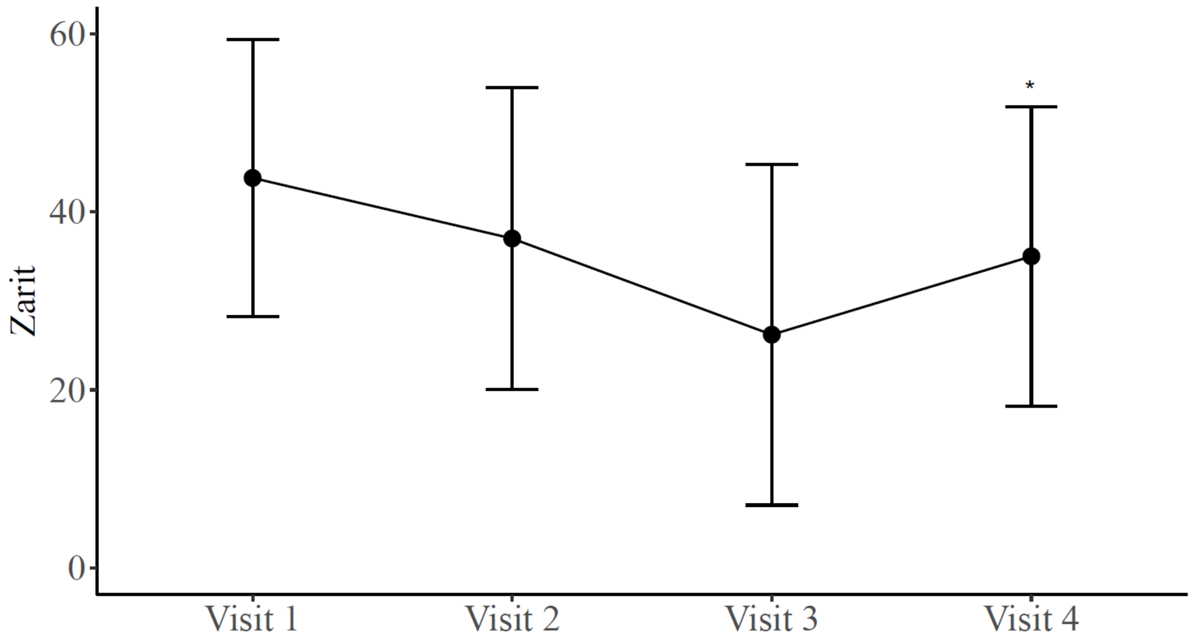

Supplement: Supplemental Material - Feasibility and Usage of a Virtual Assistant Device in Cognitively Impaired Homebound Older Adults [file sj-pdf-1-jag-10.1177_07334648251314284.pdf]
